# A non-lethal method for detection of *Bonamia ostreae* in flat oyster (*Ostrea edulis*) using environmental DNA

**DOI:** 10.1038/s41598-020-72715-y

**Published:** 2020-09-30

**Authors:** Louise von Gersdorff Jørgensen, Johan Wedel Nielsen, Mikkel Kehler Villadsen, Bent Vismann, Sussie Dalvin, Heidi Mathiessen, Lone Madsen, Per Walter Kania, Kurt Buchmann

**Affiliations:** 1 Laboratory of Aquatic Pathobiology, Department of Veterinary and Animal Science, University of Copenhagen, Stigbøjlen 7, 1870 Frederiksberg C, Denmark; 2 Aquamind, Gersonsvej 7, 2900 Hellerup, Denmark; 3 Marine Biological Section, Department of Biology, University of Copenhagen, Strandpromenaden 5, 3000 Helsingør, Denmark; 4 Institute of Marine Research, Nordnesgaten 50, Bergen, Norway; 5 National Institute of Aquatic Resources, Technical University of Denmark, Kemitorvet, Building 202, 2800 Lyngby, Denmark

**Keywords:** Infectious diseases, PCR-based techniques

## Abstract

Surveillance and diagnosis of parasitic *Bonamia ostreae* infections in flat oysters (*Ostrea edulis*) are prerequisites for protection and management of wild populations. In addition, reliable and non-lethal detection methods are required for selection of healthy brood oysters in aquaculture productions. Here we present a non-lethal diagnostic technique based on environmental DNA (eDNA) from water samples and demonstrate applications in laboratory trials. Forty oysters originating from Limfjorden, Denmark were kept in 30 ppt sea water in individual tanks. Water was sampled 6 days later, after which all oysters were euthanized and examined for infection, applying PCR. Four oysters (10%) were found to be infected with *B. ostreae* in gill and mantle tissue. eDNA purified from the water surrounding these oysters contained parasite DNA. A subsequent sampling from the field encompassed 20 oysters and 15 water samples from 5 different locations. Only one oyster turned out positive and all water samples proved negative for *B. ostreae* eDNA. With this new method *B. ostreae* may be detected by only sampling water from the environment of isolated oysters or isolated oyster populations. This non-lethal diagnostic eDNA method could have potential for future surveys and oyster breeding programs aiming at producing disease-free oysters.

## Introduction

Production of European flat oysters (*Ostrea edulis)* for human consumption has decreased from 32,995 tons in 1961 to 3120 tons in 2016 mostly due to diseases caused by the parasites *Bonamia ostreae* and *Marteilia refringens*^1^. The parasites are a main concern affecting both the production and conservation of wild *O. edulis*. Limfjorden (a fjord) in Denmark was previously recognized as a bonamiosis free zone but *B. ostreae* was detected for the first time in naïve flat oysters (*O. edulis*) from Limfjorden in 2014^2,3^. Flat oyster *O. edulis* populations have suffered high mortalities all over Europe due to bonamiosis^4^ but despite the presence of the parasite in the Danish production area (Limfjorden) no major mortalities have been reported.

*Bonamia ostreae* is a protistan parasite that belongs to the genus *Bonamia* and the phylum Haplosporidia. A spore stage, which is a characteristic for Haplosporidia, has never been demonstrated in *B. ostreae* but is present in the closely related *Bonamia perspora*^5^. Previously *B. ostreae* infected areas, fallowed for several years, quickly developed infection after reintroduction of the flat oyster^6^, and one explanation for this could involve the presence of a spore stage. Another possibility is, that the fallowed areas may never have been 100% devoid of subtidal remnant flat oyster populations. Transmission of *B. ostreae* can occur directly from oyster to oyster, indicative of a direct lifecycle, allowing a fast horizontal spread^7^. However, suspicions of intermediate hosts or vector species potentially involved in disease transmission, are based on the fact that benthic organisms, were found positive for *B. ostreae* using molecular tools^8^. *B. ostreae* has also been found in oyster larvae, and the larvae may therefore contribute to the spread of the parasite during their planktonic life^9^. Experimental infections are conducted either by cohabitation or by inoculation with purified *B. ostreae* suspensions^4^. The parasite is suspected to enter the host through the gills^10,11^, become phagocytosed by haemocytes and multiply by binary fission until the haemocyte ruptures releasing parasite stages able to infect other blood cells. The host undergoes a latent period after exposure lasting from 4 weeks to several months in which the parasite cannot be detected in the tissue by histology. *B. ostreae* is most prevalent in gills and hearts of the flat oysters^12^ and these organs are often used for sampling. Intra- as well as extra-cellular parasites are, however, also excreted with oyster faeces^4^.

Diagnosis of infection can be achieved by e.g. heart or gill imprints, histology and PCR as recommended by the World Organisation for Animal Health^13^. It is however problematic to determine mild infections of *B. ostreae* in oysters. During very mild infections or during the latent period, histological and smearing methods may not be sufficiently sensitive for detection of the parasite. Molecular analyses based on PCR using tissue DNA as template are considered more sensitive compared to light microscopy techniques^12,14–16^. Even though pieces of tissues are used for PCR techniques the likelihood of overlooking the infection is relatively low since infection is usually systemic, with infectious parasites being transported by haemocytes throughout the whole body. The main drawback of this method is that oysters have to be sacrificed or invaded if drawing haemolymph making it impossible to follow the disease progression in non-handled individuals. Environmental DNA (eDNA) released from the oysters to the environment can be detected and serve to demonstrate presence or former presence of specific organisms associated with the oysters^17–20^. Using this methodology, sampling of the organism itself is unnecessary. eDNA released from the organism to the environment can arise from faeces or other forms of secretions, sloughing off cells, small parts of tissue released to the environment or DNA leftovers from dead organisms. eDNA has a sticky nature and is known to adhere to particles of various sizes^18^. Since the parasite is transmitted from oyster to oyster it must also at some point be present in the water in a live form. Oysters infected with *B. ostreae* will discharge remnants of the parasite to the environment and detection of the eDNA in water will thereby represent a non-lethal sampling technique. The parasite DNA will be detected not only as free DNA but also from particles present in the environment.

Field studies and molecular analyses such as identification of quantitative trait loci (QTL) and resistance markers have been conducted to identify flat oyster resistance towards *B. ostreae*^21,22^. It is relevant to investigate if selective breeding of resistant oysters is feasible in order to restore this economically and environmentally important species. In order to select resistant and healthy oysters for breeding purposes, the *B. ostreae* infection status of the oyster must be determined by non-lethal techniques.

We here present a method for application of eDNA detection in the oyster environment to determine presence or absence of *B. ostreae* in *O. edulis* from laboratory stocks and wild populations. With such a technique surveillance, diagnosis, selection of disease-free oysters and management in cultured oyster stocks may be achievable.

## Materials and methods

### Oysters

**Figure 1:**
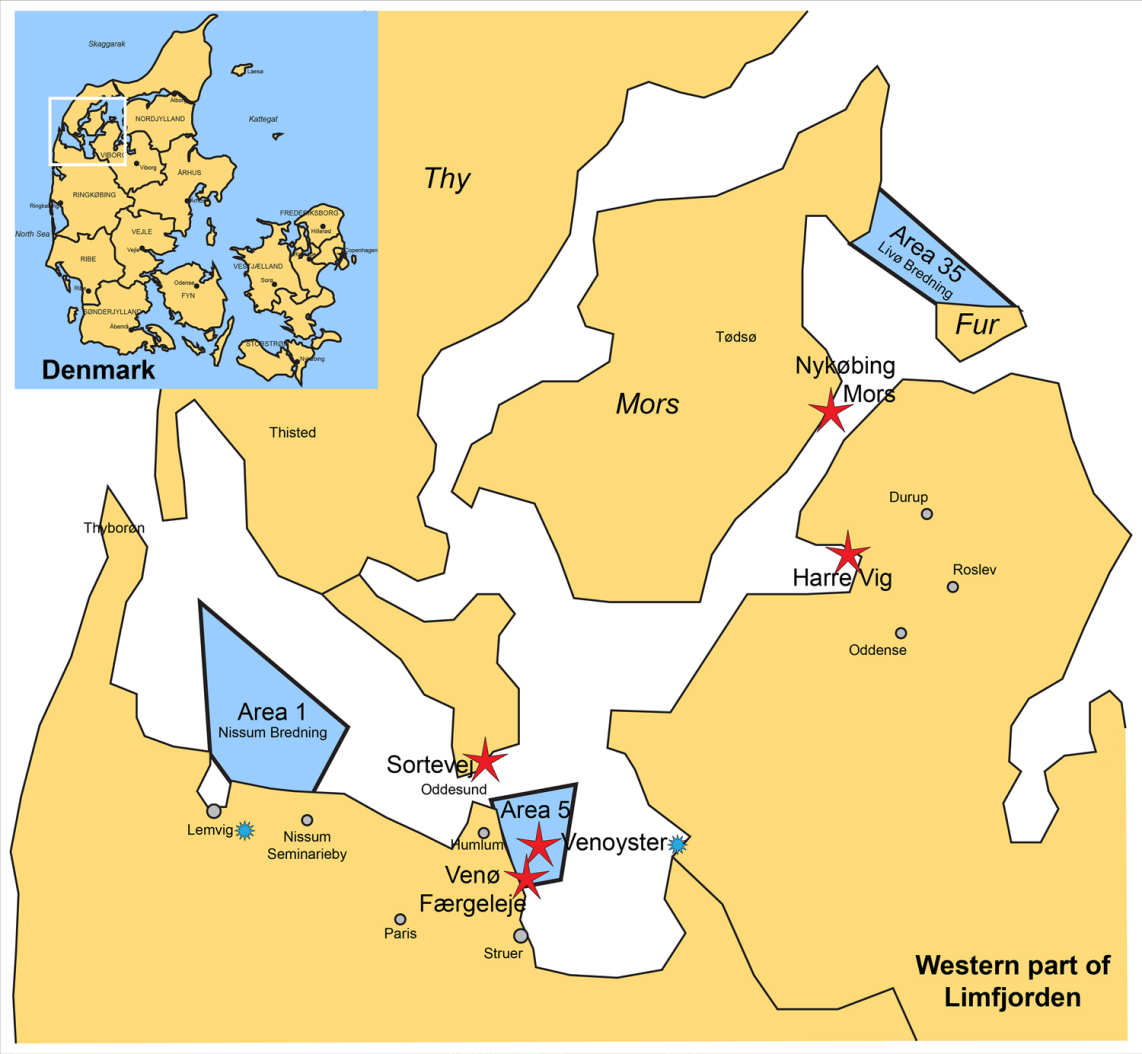
Map of sampling locations of flat oyster *Ostrea edulis* in the western part of Limfjorden, Denmark. The small map in the upper left corner shows the location of Limfjorden in Denmark. From area 1 and 35, oysters were collected and kept in the laboratory for individual isolation for 6 days and subsequent analyses for *Bonamia ostreae* DNA and eDNA in the oysters and in the water, respectively. The red stars indicate locations of oyster and water sampling in the field for DNA and eDNA detection of the parasite *B. ostreae*. The blue star specifies that the oysters at the oyster farm Venø Seafood ApS (Venoyster) originated from Lemvig. Map modified from *World of Maps Editable Clip Art Download Collection*.

Forty oysters in total were caught in 2019 in two areas in Limfjorden, Denmark (area 1 and 35, Fig. 1, 20 oysters at each location) and brought to the laboratory of Marine Biological Section, University of Copenhagen, Helsingør, where the oysters from each location were kept in two separate tanks with running and aerated seawater (≈30 ppt, 10–12 °C). To decontaminate the seawater before use, it passed through a sand filter, proteins were removed by foaming the water and it was UV and ozone treated. The seawater originated from Øresund (stretch of water between Denmark and Sweden) from a depth of 20 m. In Øresund, flat oysters are absent and during the investigation our oysters were the only oysters present at the Marine Biological Section. The oysters were fed *Rhodomonas salina* every second week. The 40 oysters were, at two different time points (20 oysters each time), brought to the Laboratory of Aquatic Pathobiology, University of Copenhagen, Frederiksberg C and kept in separate static tanks with 2 L of seawater (≈30 ppt). After 6 days, the water was collected and the oysters were euthanized and sampled. *B. ostreae* negative oysters were obtained from Tony Legg, Jersey Sa Farms, Jersey, UK and DNA from an oyster positive for the parasite (used as a positive control) was obtained from DTU Aqua, Technical University of Denmark. The positive oyster originated from area 5 (Fig. 1) in Limfjorden, where it had been sampled in November 2016 and had been found positive for *B. ostreae* by histology, heart imprints and PCR. DTU Aqua serves as National Reference Laboratory for fish, crustacean and mollusc diseases. The map in Fig. 1 was created from *World of Maps Editable Clip Art Download Collection* purchased from Gumroad, USA.

### Water and tissue sampling

All the water surrounding each oyster, including faecal debris, was collected in clean autoclaved 2 L bluecap glass bottles and kept at 4 °C until filtration, which was conducted within a maximum of 24 h. Tissue was sampled from the gills and the mantle of the oysters and pieces kept in 70% ethanol for PCR analyses and 10% neutral buffered formalin (Hounisen, Denmark) for histology.

### Filtration

Two litres of water with sediment from the isolated oysters from area 1 and 35 and 3 × 1 L of water from the field sampling were filtered through a 112 µM pore filter and the filtrate was kept for further filtration through filters with pore sizes of 10 µM (CMF coated acetate, Advantec) followed by 1.2 µM (mixed cellulose ester, Frisenette). Water from 10 oysters from area 1 was only filtered through 112 µM and 1.2 µM filters. Filters were immediately frozen at -20 °C in individual plastic bags until further analysis. The 10 µM filters retain oyster cells, which can carry the parasite and other particles potentially holding *B. ostreae* eDNA. The 1.2 µM filters filter out the parasites, which are 2–4 µM of size and other particles potentially holding *B. ostreae* eDNA. All tables and material used were cleaned with water and soap, ethanol and hydrogen peroxide (H_2_O_2_) between samples to clear DNA residues.

### Sampling in Limfjorden

Oysters and water samples were collected from 5 different locations in Limfjorden (Fig. 1) in 2019 in late spring. From the oyster farm “Venø Seafood ApS”, gill and mantle tissues from 5 oysters were sampled for PCR and histology. One litre of water was sampled in triplicate at the oyster farm and immediately filtered through a metal sieve with a pore size of 112 µM. The filtrate was then filtered through filters with pore sizes of 10 µM and subsequently 1.2 µM. The 3 × 2 filters were frozen at − 20 °C for subsequent analysis. Five flat oysters were collected from each of the remaining three sampling spots (Sortevej, Venø Færgeleje and Harre Vig, Fig. 1) by snorkelling and collecting by hand. At one location (Nykøbing Mors), the water was too turbid to collect oysters and only water was collected. Oysters were brought to the shore where gill and mantle were sampled for PCR and histology. Three water samples, each 1 L, were acquired in the immediate water surroundings of sampled oysters. Here, bottles were submerged and the lid was removed when the bottles were near flat oysters at the bottom substrates. Each bottle was contained in separate closed plastic bags. Water was kept at room temperature for up to 10 h before filtration (described above) was possible. After filtration, the 30 (5 × 3 × 2) filters were immediately frozen at − 20 °C.

### PCR

DNA from tissues were purified with QIAamp DNA mini kit (Qiagen, Denmark) following manufacture’s protocol (eluted in 200 µL) and measured on nanodrop 2000 (Saveen & Werner APS, Sweden) to check concentration and quality. Purification was conducted in a room dedicated to this and PCR was set up in a different room. The primer pair for the PCR consisted of a forward primer for 18S and a reverse primer for ITS1 (Table 1). The size of the expected PCR sequence was 343 bp. PCR conditions were: 10 µM primers, 1.5 mM MgCl_2_, 10 × reaction-buffer, 10 mM dNTP mix, taq DNA polymerase (Biotaq, cat.no. BIO-21060, Saveen & Werner), nuclease free H_2_O, template using the following cycling parameters: 2 min pre-denaturation at 95 °C, 45 cycles of 30 s of denaturation at 95 °C, 30 s of annealing at 60 °C and finally 7 min of post elongation at 72 °C. All PCR products were analysed on a 1.5% agarose gel and visualized with ethidium bromide. A positive control (DNA from a *B. ostreae* positive oyster), a negative control (DNA purified from *B. ostreae* negative oysters) and no template controls were included. Furthermore, the obtained PCR products were confirmed by sequencing at Macrogen, Korea.

**Table 1:** Primers, probes and references for PCR and qPCR assays used in this study. All the assays had efficiencies within 100% ± 5% and an annealing temperature of 60 °C.

Gene Organism		Sequence read from 5′ end to 3′ end	Reference
18S *B.* o*streae*	Forward primer: Reverse primer: Probe:	CCCGGCTTCTTAGAGGGACTA ACCTGTTATTGCCCCAATCTTC FAM-CTGTGTCTCCAGCAGAT-BHQ1	Marty et al. (2006)^23^
ITS1 *B.* o*streae*	Forward primer: Reverse primer: Probe:	CCCTGCCCTTTGTACACACC TCACAAAGCTTCTAAGAACGCG FAM-GGTGAATTAGGTGGATAAGAGCGCT-BHQ1	Corbeil et al. (2006)^12^
ELF 1α *O. edulis*	Forward primer: Reverse primer: Probe:	GTCACGGACAGCAAAACGTC TCGATTGCCACACTGCTCAT FAM-GGTGAATTAGGTGGATAAGAGCGCT-BHQ1	Present study
IAC IAG52B	Forward primer: Reverse primer: Probe:	CCAGTGTATCGCCTGTCAGG ACTGGGTGAAGGTGGGAGAT CY5-GGCGGTGCCGGCAGGACACAGG-BHQ2	Present study

An internal amplification control assay (IAC) designed in the present study consisted of a plasmid with an artificial IAG52B construct and a CY5 labelled probe. Thus, this IAC was used in our assays using FAM labelled probes to detect inhibition in each sample.

### qPCR

DNA from whole filters was purified with the DNeasy PowerWater Kit (cat.no. 14900-100-NFm, QIAGEN, Denmark) according to the manufacturer (eluted in 50 µL), validated on a nanodrop, and subsequently stored at − 20 °C. Like for PCR, purification was conducted in a dedicated room and qPCR was set up in a different room. Filter tips were used at all time points and decontamination procedures were carefully and regularly conducted. The qPCR method was chosen due to high sensitivity and low concentrations of eDNA (30–280 ng/µL) from the filters. Our qPCR assays included already published *Bonamia* sp. specific primer sets and probes for ITS1 and 18S (Table 1) and we furthermore designed primers for ELF 1α from *O. edulis* as previously described^24^*.* A TaqMan Environmental Master Mix 2.0 was used for the qPCR. All qPCR reactions except for ELF 1α were run twice and the Ct cut off value was set at 37. qPCR reactions were as follows: 5 µL sample, 10 µM primers and 5 µM probes, TaqMan Environmental Master Mix 2.0 (cat.no. 4396838, Thermo Fisher Scientific, Denmark). The qPCR conditions consisted of 10 min of pre-denaturation at 95 °C, 45 cycles of 15 s denaturation at 95 °C and 1 min elongation at 60 °C. A positive and a negative control (as described above for PCR) for *B. ostreae* and three no template controls were included in all runs.

### Internal amplification control assay (IAC)

This assay was developed to detect potential inhibition in environmental water samples as well as in tissue samples. The assay detected expression of a plasmid (Supplementary Fig. S1) containing a gene encoding IAG52B (a surface protein of the freshwater fish parasite *Ichthyophthirius multifiliis*)^25^. Based on the amino acid sequence, an artificial nucleotide sequence using codons optimized for expression in rainbow trout (*Oncorhynchus mykiss)* was designed and inserted into the plasmid pcDNA. Thus, this target sequence of IAG52B does not exist in the environment or in any free-living species. The plasmid was added to all eDNA and tissue samples plus no template controls together with primers and a CY5 labelled TagMan probe targeting the artificial sequence. Inhibition was defined if Ct values differed more than three cycles from the no template controls^26^.

### Sensitivity assay

Gill tissue from a *B. ostreae* positive oyster was homogenized in PBS using a pistil in an Eppendorf tube. This homogenate was subjected to 10 × serial dilution up to 10,000 ×. Volumes of 100 µL of each dilution and the undiluted homogenate were added to 100 ml of phosphate buffered saline (PBS) in triplicate (5 × 3 triplicates). These samples and a negative control (100 ml PBS) were filtered through filters with a pore size of 1.2 µM. DNA was purified from the filters with the DNeasy PowerWater Kit and eluted in 50 µL. DNA was used for qPCR analyses together with triplicates of DNA from 100 µL of the crude homogenate, which was purified with QIAamp DNA Mini Kit (cat.no. 51306, Qiagen, Denmark) and eluted in 50 µL.

### Histology

After 24 h in fixative the oyster tissues were transferred to 70% ethanol and kept at 4 °C until they were embedded in paraffin. Consecutive sections of 4 µM were cut on a microtome (Leica RM2135) and deparaffinized before Hematoxylin and Eosin staining (H&E). Slides from all positive oysters were examined independently by two researchers.

### Data analyses

For qPCR, our interpretation of results was as follows: if one run out of two was positive the sample was considered suspicious of containing *B. ostreae* DNA and if both runs had Ct values below 37 the oyster was considered positive for *B. ostreae* DNA*.*

## Results

### *B. ostreae* in flat oysters

**Figure 2:**
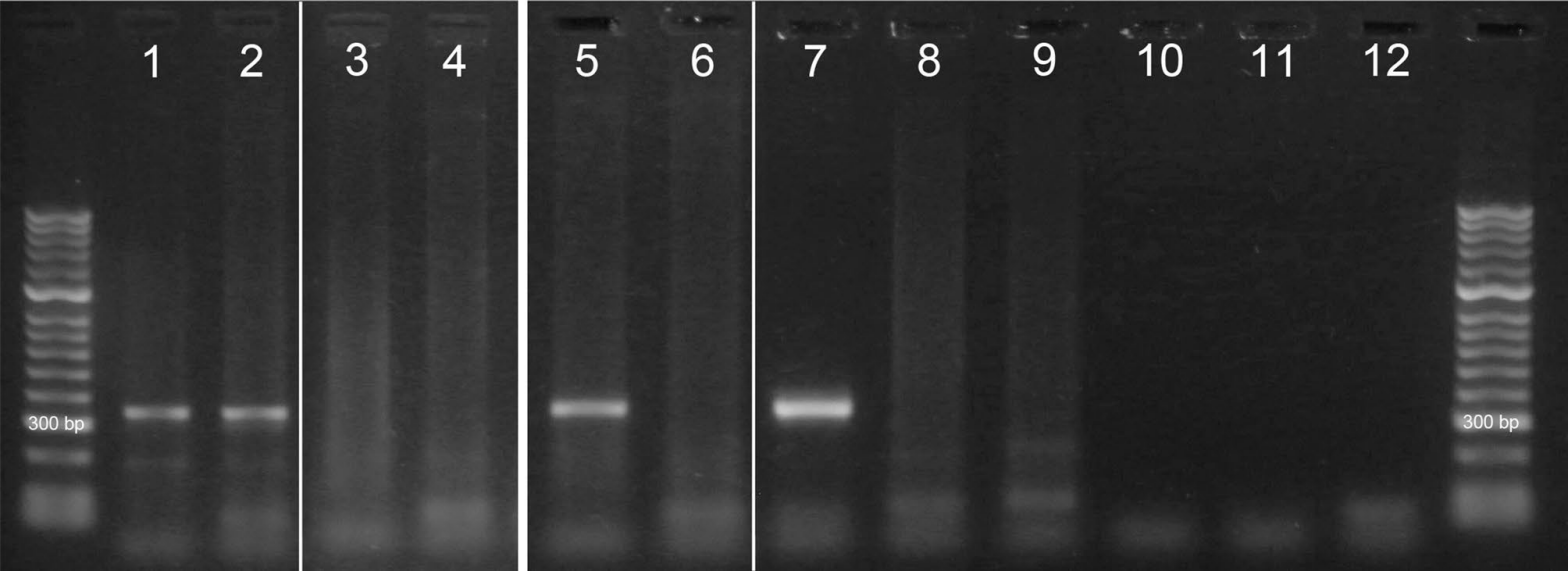
Regular PCR of a product covering parts of 18S and ITS1 of *Bonamia ostreae* using gill and mantle tissue from the oysters. Lane 1–2 gill tissue from two flat oysters positive for *B. ostreae*. Lane 3–4 gill tissue from flat oysters from Limfjorden negative for *B. ostreae*, lane 5 mantle tissue from a third oyster positive for *B. ostreae*, lane 6 mantle tissue from a flat oyster negative for *B. ostreae*, lane 7 positive control sample, lane 8–9 negative control samples from *B. ostreae* free oysters from Jersey, lane 10–12 no template control reactions. White lines indicate where parts from the same gel (Supplementary Fig. S2) were merged.

The first 20 oysters (10 from area 1 and 35, respectively), brought to the laboratory in Copenhagen from Helsingør, were examined and were all negative for *B. ostreae* from gill and mantle tissue samples. From the following 20 oysters isolated in the laboratory (10 from area 1 and 35, respectively), 4 were positive (three from area 35 and one from area 1, Supplementary Table S1) from regular PCR on either gill or mantle tissue or both (Fig. 2, Supplementary Fig. S2). Sequencing of the positive control and a positive oyster confirmed that the bands represented DNA from *B. ostreae*. From the 20 oysters directly sampled in the field, only one oyster was positive for the parasites in the gill tissue (Supplementary Table S2).

### *B. ostreae* from water samples

**Figure 3:**
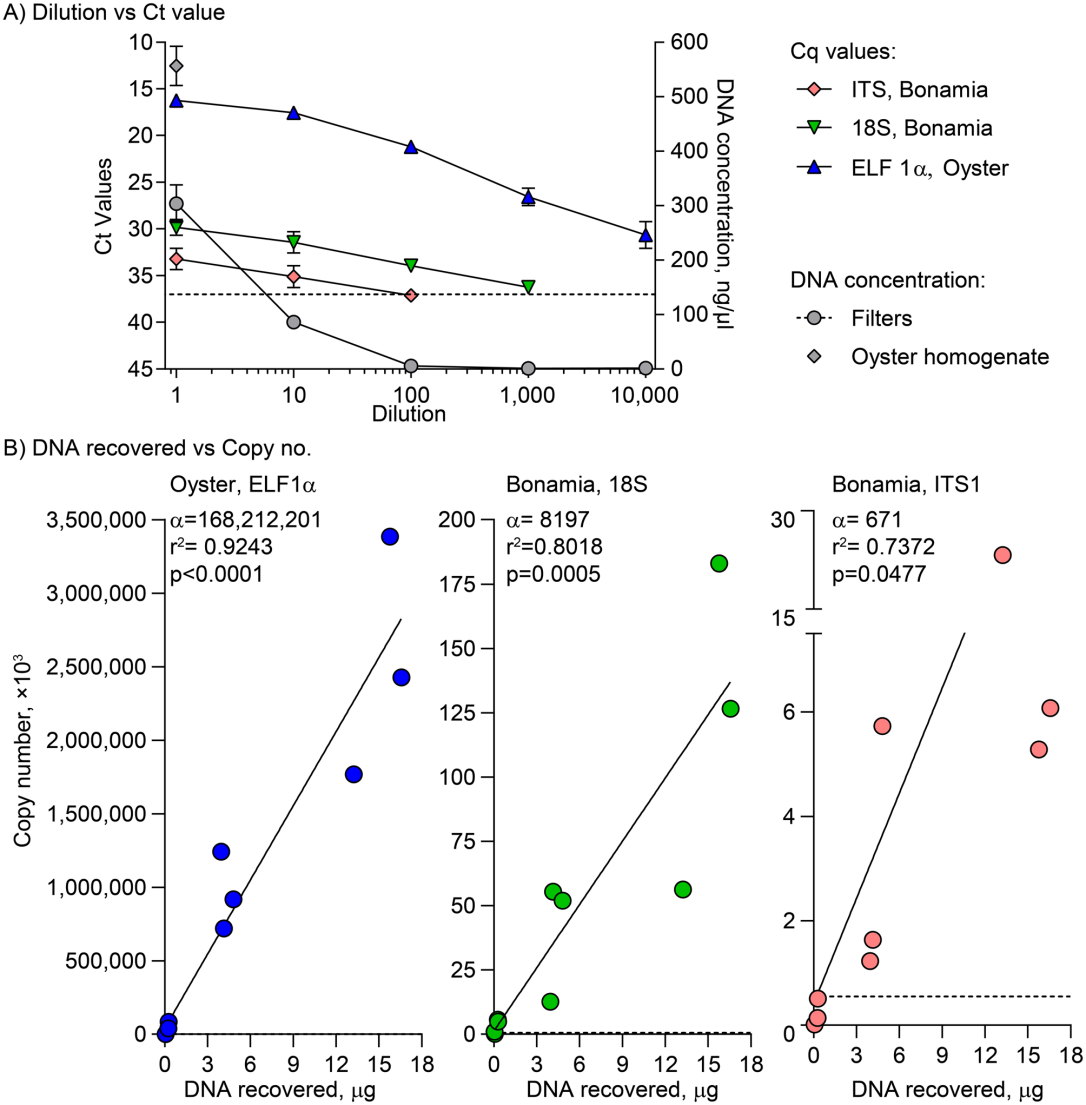
qPCR sensitivity of *Bonamia ostreae* and flat oyster (*Ostrea edulis*) DNA. 100 µL of *B. ostreae* positive oyster homogenate in 100 ml of PBS was subjected to a tenfold dilution series ranging from 1 to 10,000. **(A)** The relationship between the concentration of DNA from filtered spiked PBS samples and qPCR results. The Ct values for the ITS1 and the 18S genes from *B. ostreae* as well as the ELF 1α gene from *O. edulis* and the concentration of DNA from filters are indicated. The undiluted case in PBS showed 55% recovery of DNA compared to the amount of DNA from the homogenate. **(B)** The x-axis presents the amount of genomic DNA from the samples. The y-axis presents the copy number of the genes based on standard curve calculations. The slope α shows that the amount of ELF 1α DNA per µg DNA recovered from the filters was much higher than DNA from the parasites. The horizontal stipulated lines at 550 copies indicates the threshold Ct value of 37.

Water samples from the first 20 *B. ostreae* negative oysters were analysed for false positives of *Bonamia* by qPCR^12^ and were all found to be negative (data not shown). Only the 10 µM filters were used here and since 18S appeared to be more sensitive compared to ITS1 (Fig. 3), it was the only gene analysed. Analyses of the eDNA from water samples from the following 20 individually isolated oysters revealed that three samples out of 10 from area 35 (Table 2) included *B. ostreae* eDNA and that three samples were under suspicion (one out of two qPCR replicates turned out positive) of including DNA from the parasite. Filters of different pore sizes were analysed separately. The water sample results from oyster 11 and 12 in Supplementary Table S1 were obtained from the two oysters represented in lane one and two in Fig. 2. From area 1, only one positive and 1 sample under suspicion were identified (Supplementary Table S1). The 4 positive water samples matched the oysters, which were found positive for the parasite in the gill and/or mantle tissue by regular PCR. From the eDNA obtained from water filtration in the field no sample turned out positive. Of the 30 × 2 qPCR runs using the field water samples (3 × 1 L/location, 5 locations, 2 filters/sample) one single sample, however, was under suspicion of including *B. ostreae* eDNA (Supplementary Table S2).

**Table 2:** The average Ct values from qPCR results analysing genes 18S and ITS1 from *Bonamia ostreae* from 10 water samples (2L) from tanks with flat oysters from area 35.

Pore size of filter		Oyster number									
		1	2	3	4	5	6	7	8	9	10
1.2 µM	ITS1	–	35.2	–	–	–	–	–	–	–	–
	18S	–	33.2	–	–	–	–	–	–	–	*35.9*
	ELF1α	31.9	28.2	29.8	32.8	32.4	30.9	32.8	30.3	–	33.3
10 µM	ITS1	–	32.5	–	–	–	–	–	–	36.3	–
	18S	35.3	34.7	–	*33.8*	*31.5*	–	–	–	34.2	–
	ELF1α	27.8	25.5	26.8	27	29.4	28.1	28.2	27.8	26.3	27.3

Water samples were filtrated using filters with pore sizes of first 10 µM and then 1.2 µM. Threshold was set to 37, thus all Ct values below 37 were regarded as positive for *B. ostreae*. “No Ct” values are indicated by a minus (−). The Ct value of the positive control was ~ 30 for ITS1, ~ 28 for 18S and ~ 29 for ELF 1α. Values in italics represent samples, where one qPCR reaction resulted in “No Ct” value and the other a Ct value below 37. These samples were regarded as under suspicion of including *B. ostreae* eDNA.

### Sensitivity assay

A sensitivity assay was included in this study to estimate how much DNA is lost by the filtration method as well as how much DNA is necessary to obtain solid data. Results showed that with a dilution of 1:1000 (73 ng DNA recovered on average in 50 µL) the signal disappeared by exceeding the Ct value of 37 (Fig. 3). *B. ostreae* ITS1 was traceable at a maximum dilution of 1:100. The slopes (α) indicate that the oyster ELF 1α was approximately 20,000 and 250,000 times more prevalent than *B. ostreae* 18S and ITS1, respectively; *B. ostreae* 18S was about 12 times as prevalent as *B. ostreae* ITS1. The recovery of DNA from 100 µL of homogenate poured into 100 ml PBS and subsequently filtered was 55%.

qPCR on the positive control sample resulted in lower ELF 1α DNA levels and higher 18S and ITS1 *B. ostreae* DNA levels compared to infected oysters investigated here. This indicates that the flat oysters from this study had lower *B. ostreae* infections than the positive control.

### Internal amplification control

No primer/dimers and unspecific products were observed when the selected primer combination was tested using SYBR green qPCR and melting curve analysis. A 10 × dilution series of the plasmid starting from 2.8 × 10^10^ to 2.8 copies was performed. Log-linear regression of the number of copies against the obtained Ct values resulted in a line with an r^2^ of 0.9775, a slope of -3.3377 and an intercept of 37.98 (Supplementary Fig. S3). The efficiency was 99.3% in the range from 28 to 28 × 10^9^ copies.

### Inhibition

No inhibition was observed in the water samples (data not shown) as the difference to the control sample (H_2_O) was less than three Ct values.

### Histology

Oysters detected as positive for *B. ostreae* by PCR were analysed by histology but the parasite was not found in the sampled gill or mantle tissues.

## Discussion

The European populations and production of flat oyster *O. edulis* have decreased markedly over the last four decades^4^. The parasitic disease bonamiosis caused by the protist *B. ostreae* is a major reason for the decline. However, breeding of *B. ostreae* resistant oysters to restore the production may be one way to mitigate the problem. A brood stock of healthy oysters is a prerequisite for such a production and a non-lethal detection method to select disease-free oysters is desirable. This way cultivated or wild populations will be minimally disturbed or reduced. The present study evaluates a non-lethal water sampling method detecting *B. ostreae* DNA (eDNA) in water containing *B. ostreae* infected European flat oyster. In laboratory experiments, we showed that parasite eDNA could be detected in seawater from tanks containing infected oysters following an incubation period of 6 days. The water samples included all material released from the oysters. Some of the field water samples were obtained from infected localities (we found one positive oyster) but no parasite eDNA was recovered. This may partly be explained by the fact that faeces from oysters including particles larger than 1 µM^27^ and microbial DNA from the water column settle and accumulate in the sediment^18,19^ and were therefore not sampled.

It is difficult to provide an exact diagnosis of *B. ostreae* and in general a likelihood estimation of infection is used following application of methods including imprints, histology and/or PCR. However, for all of these techniques it is known that low infection levels can go unnoticed^13^. The eDNA method described in the present work may be as sensitive as classical PCR since we from the oyster tanks obtained *B. ostreae* eDNA fragments from all oysters confirmed positive for the parasite by PCR. Further comparative studies should evaluate the sensitivity in relation to the most applied and recommended molecular tools but it is hypothesized that the eDNA method may have a higher sensitivity because cellular debris from e.g. haemocytes carrying parasite DNA and other necrotic oyster cells with parasites are excreted with faeces and accumulate in the oyster tank—even during mild infections. Recovery of eDNA by filtration of water samples alone (without sediment) may, on the other hand, be less sensitive than classical PCR on tissues since we showed that DNA signals are reduced by around 55% when the DNA is diluted in water (Fig. 3). Results obtained here, provide us with a preliminary correlation of how eDNA relates to infection profiles in oysters but further investigations are warranted to reach a high level of insight. The results can, nonetheless, be used as a first step in assay development of a new front-line molecular tool using eDNA for detection of *Bonamia* and for use in other host–pathogen systems.

It is generally agreed that eDNA sticks to particles^28,29^ and may at least partly be held back on the filters used. In the present study filters with a pore size 1.2 µM would catch the parasites (size range 2–4 µM) or maybe their mitochondria and other particles^18^. We do not recommend using smaller pore sizes due to observed clogging of these filters prolonging sampling time significantly.

The main purpose of this study was to develop a non-lethal method for selection of non-infected oysters to be used for disease-free rearing. The procedures described should find application for the purpose but as it cannot be guaranteed that very mild infections are detectable—also using this method—we recommend to combine it with strict quarantine procedures. With our non-lethal sampling method, the number of sacrificed animals will be reduced and disease-free oysters can furthermore be identified and dedicated as parental animals.

For field applications, further comparative investigations must be conducted and until a more appropriate field technique has been described we recommend standard identification methods (OIE, 2019). Refinement of the field methodology could include sampling of the water as well as sediment and development of even more sensitive techniques e.g. amplification of eDNA before application for qPCR. With an improved technique, costs associated with diagnosis and surveillance would likely be reduced since for example histology is very resource demanding. Furthermore, groups of oysters could be diagnosed with one water sample reducing costs compared to sampling every oyster for PCR and histology. The method has potential following further optimizations as a surveillance tool representing a non-laborious and very cost-efficient method and predictions on the spread of bonamiosis and identification of areas in danger of exposure may even be possible.

To implement this non-lethal eDNA technique as a surveillance tool for bonamiosis it needs additional refinement. Investigations on the period of isolation and the necessary amount of water to filter should be assessed. Our study was limited by the low number of infected animals and further sampling would strengthen the results. Additionally, to test sensitivity and progress of disease, taking out water samples from oysters with bonamiosis and newly infected oysters over a period of time e.g. 6 months could uncover sensitivity issues and threshold values for when the oysters die from disease. We conducted such an investigation but out of the 8 oysters we collected from the field and followed for 5 months (sampling every 4 weeks) none of them showed infection when we sampled DNA from the tissue at the end of the period. The bonamiosis progression in an oyster as well as real-time transmission and seasonal variation may be followed closely with this new methodology. Besides monitoring *B. ostreae* we have shown that we also can monitor flat oyster in water samples, which may be used for surveillance of oyster stock- and wild populations. In all laboratory water samples and in 13 out of 15 water samples from the field study *O. edulis* eDNA was detected. Our results furthermore indicated that the positive oysters were mildly infected, which was supported by the absence of parasites in histological analyses.

We conclude that the water filtration method detecting *B. ostreae* eDNA represents a promising tool to significantly improve diagnosis of bonamiosis in flat oyster. A further perspective of the technique is the application for detection of a wide range of other diseases in oysters as well as mussels or other aquatic animals.

## Supplementary information

Supplementary Information.

## Data Availability

No restrictions.
