# Supplementary material for: A non-lethal method for detection of Bonamia ostreae in flat oyster (Ostrea edulis) using environmental DNA
Source: Sci Rep. 2020 Sep 30;10:16143. doi: 10.1038/s41598-020-72715-y (PMC7527985; doi:10.1038/s41598-020-72715-y)
Supplement: Supplementary file 1 — Supplementary Information. [file 41598_2020_72715_MOESM1_ESM.pdf]

A non-lethal method for detection of *Bonamia ostreae* in flat oyster (*Ostrea edulis*) using environmental DNA

Louise von Gersdorff Jørgensen[1], Johan Wedel Nielsen[2], Mikkel Kehler Villadsen[2], Bent Vismann[3], Sussie Dalvin[4], Heidi Mathiessen[1], Lone Madsen, Per Walter Kania[1] and Kurt Buchmann[1]

Supplementary materials

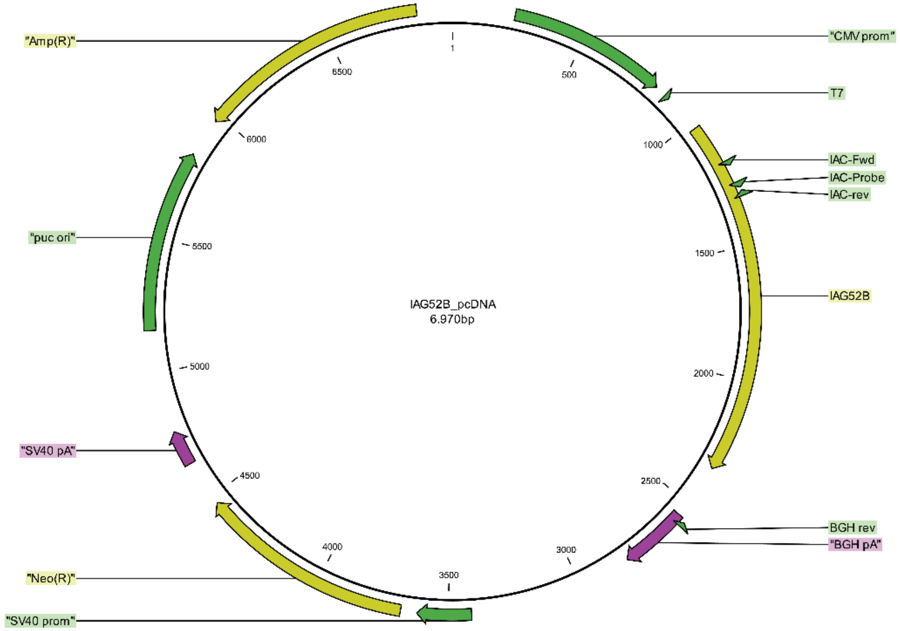

Supplementary Figure S1. **IAC plasmid design.** Outline of a plasmid, pcDNA, containing an artificial nucleotide sequence encoding a surface protein, IAG52B, from the fish parasite *Ichthyophthirius multifiliis* with codons optimized for expression in rainbow trout (*Oncorhynchus mykiss)* used in the *Internal Amplification Control* assays.

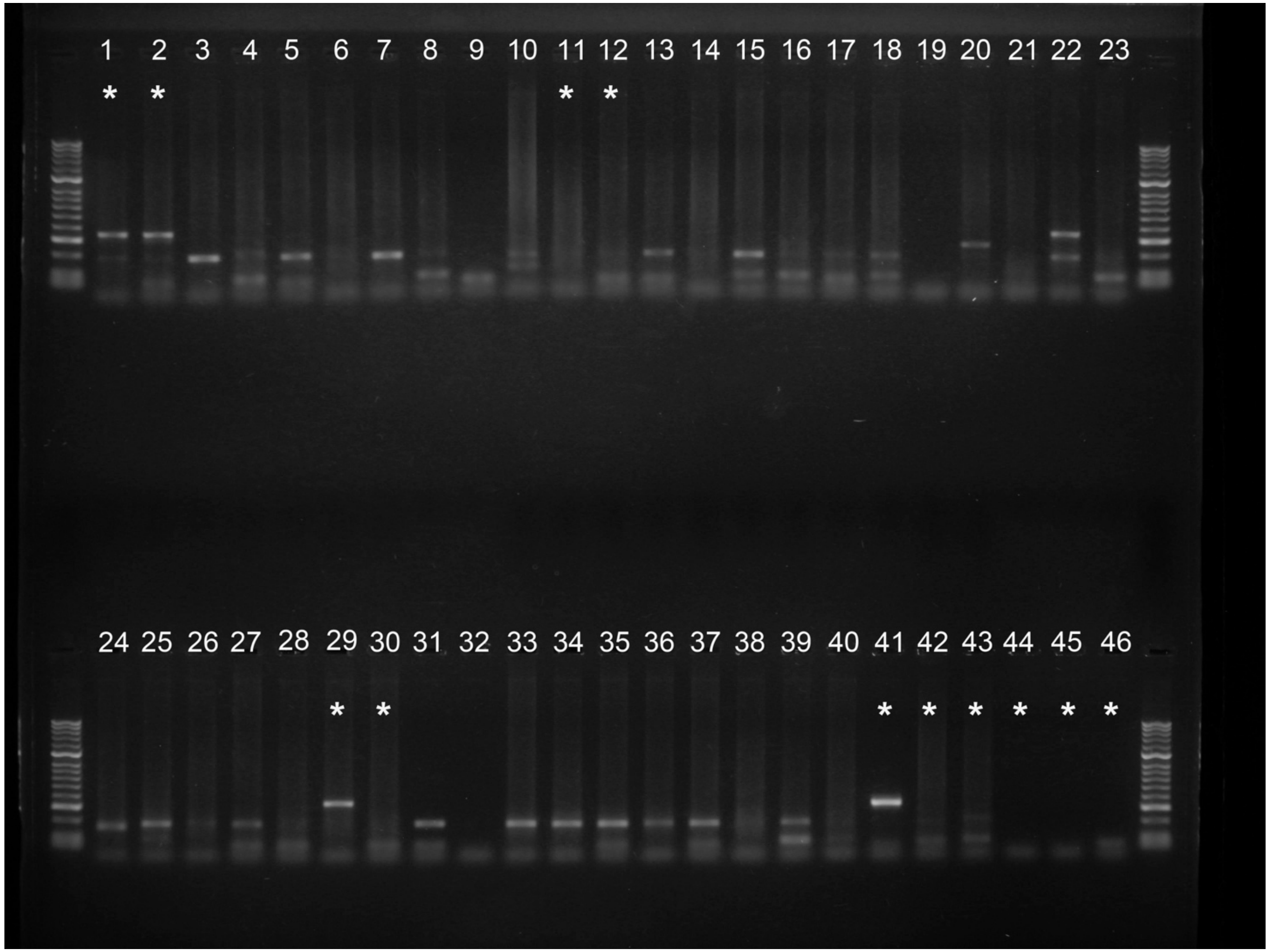

Supplementary Figure S2. Regular PCR of a product covering parts of 18S and ITS1 of *B. ostreae* using gill and mantle tissue from oysters. Expected size of product = 343 bps. Lane 1-8; 10-18 and 19-20 gill tissue from flat oysters, lane 21-30 and 33-40 mantle tissue from oysters, lane 41 positive control sample, lane 42-43 negative control sample from *B. ostreae* free oysters from Jersey, lane 9, 19, 31-32 and 44-46 no template control reactions. Lane 1-2, 11-12, 29-30, 41-46 (marked with *) equals lane 1-2, 3-4, 5-6 and 7-12 in Figure 2, respectively.

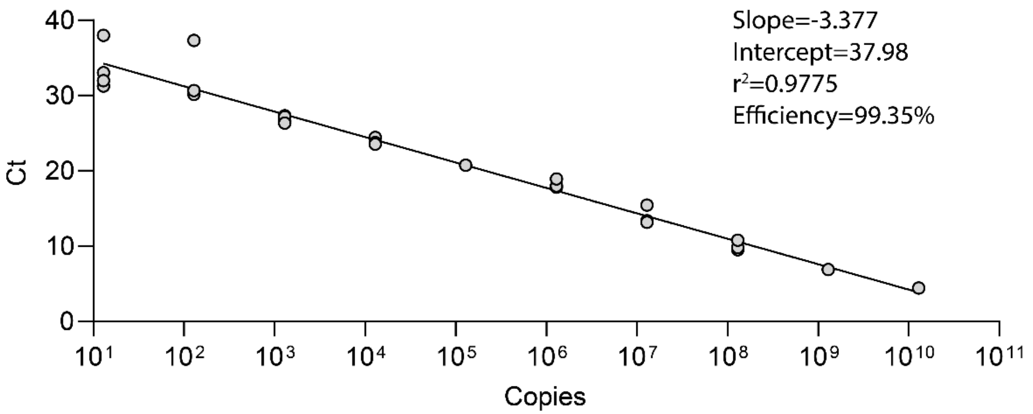

Supplementary Figure S3. **Standard curve for the Internal Amplification Control using a probe based TagMan assay**. To obtain a standard curve, a plasmid containing an artificial nucleotide sequence encoding the IAG52B of *Ichthyophthirius multifiliis* designed for expression in a eukaryote, was used in a 10x dilution series. The copy number of a given Ct value is calculated by $10^{(\text{Ct-intercept})/\text{slope}}$.

			Area 1										Area 35									
**Oyster Number**			1	2	3	4	5	6	7	8	9	10	11	12	13	14	15	16	17	18	19	20
**PCR, DNA**	ITS-18S	Mantle	-	-	-	-	-	-	-	-	-	P	-	P	-	-	-	-	-	-	P	-
		Gills	-	-	-	-	-	-	-	-	-	P	P	P	-	-	-	-	-	-	P	-
**qPCR, eDNA**	1.2 µm	ITS1	-	-	-	-	-	-	-	-	-	35.2	-	35.2	-	-	-	-	-	-	-	-
		18S	-	-	-	-	-	-	*34.5*	-	-	34.2	-	33.2	-	-	-	-	-	-	-	*35.9*
		ELF1α	29.1	29.3	31.4	27.7	30	30.8	30.6	27.3	30.5	28.17	31.9	28.2	29.8	32.8	32.4	30.9	32.8	30.3	-	33.3
	10 µm	ITS1											-	32.5	-	-	-	-	-	-	36.3	-
		18S											35.3	34.7	-	*33.8*	*31.5*	-	-	-	34.2	-
		ELF1α											27.8	25.5	26.8	27	29.4	28.1	28.2	27.8	26.3	27.3

Supplementary Table S1. An overview of the PCR and qPCR (Ct values) results of tissues and water samples from the second sampling from 20 oysters originating from two different areas of Limfjorden, Denmark but kept in the laboratory before sampling. PCR was conducted on mantle and gill tissues and qPCR on environmental DNA (eDNA) obtained from water samples from the oyster tanks. qPCR; threshold was set to 37, thus all Ct values below 37 were regarded as positive for *Bonamia ostreae*. “No Ct” values are indicated by a minus (-). Values in gray italics represent samples, where one qPCR reaction resulted in “No Ct” value and the other a Ct value below 37. These samples were regarded as under suspicion of including *B. ostreae* eDNA. P = conventional PCR positive for *B. ostreae*.

Location in Limfjorden, Denmark																											
			Venoyster					*Harbor at Venø*					*Sortevej 3*					*Harevig*					*Nykøbing Mors*				
Tissue samples (DNA)																											
Oyster number			1	2	3	4	5	6	7	8	9	10	11	12	13	14	15	16	17	18	19	20					
**PCR**	ITS-18S	Mantle	-	-	-	-	-	-	-	-	-	-	-	-	-	-	-	-	-	-	-	-					
		Gills	P	-	-	-	-	-	-	-	-	-	-	-	-	-	-	-	-	-	-	-					
Water samples (eDNA)																											
			1	2	3			4	5	6			7	8	9			10	11	12			13	14	15		
**qPCR**	1.2 µm	ITS1	-	-	-			-	-	-			-	-	-			-	-	-			-	-	-		
		18S	-	-	-			-	-	-			-	-	-			-	-	-			-	-	-		
		ELF1α	24	27,2	30,8			34,3	30,1	33,5			30,5	33,9	34			33,4	36,1	-			-	36,7	-		
	10 µm	ITS1	-	-	-			-	-	-			-	-	-			-	-	-			-	-	-		
		18S	-	-	-			-	-	-			*36.1*	-	-			-	-	-			-	-	-		
		ELF1α	34,2	27,0	33,2			31,6	32,9	-			-	34,0	32,6			31,4	37,1	35,1			-	36,8	-		

Supplementary Table S2. An overview of the PCR results of 20 oysters sampled from four different locations and qPCR (Ct values) results from 15 water samples collected at five different locations in Limfjorden, Denmark. PCR was conducted on mantle and gill tissues and qPCR on environmental DNA (eDNA) obtained from the water samples. qPCR; threshold was set to 37, thus all Ct values below 37 were regarded as positive for *Bonamia ostreae*. "No Ct" values are indicated by a minus (-). Values in gray italics represent samples, where one qPCR reaction resulted in "No Ct" value and the other a Ct value below 37. These samples were regarded as under suspicion of including *B. ostreae* eDNA. P = conventional PCR positive for *B. ostreae*.
